# An Adagio for Viruses, Played Out on Ancient DNA

**DOI:** 10.1093/gbe/evad047

**Published:** 2023-03-17

**Authors:** Toni de-Dios, Christiana L Scheib, Charlotte J Houldcroft

**Affiliations:** Institute of Genomics, University of Tartu, Estonia; Institute of Genomics, University of Tartu, Estonia; St. John's College, University of Cambridge, United Kingdom; Department of Genetics, University of Cambridge, United Kingdom

**Keywords:** paleogenomics, ancient biomolecules, virology, aDNA, population genomics, evolution

## Abstract

Studies of ancient DNA have transformed our understanding of human evolution. Paleogenomics can also reveal historic and prehistoric agents of disease, including endemic, epidemic, and pandemic pathogens. Viruses—and in particular those with single- or double-stranded DNA genomes—are an important part of the paleogenomic revolution, preserving within some remains or environmental samples for tens of thousands of years. The results of these studies capture the public imagination, as well as giving scientists a unique perspective on some of the more slowly evolving viruses which cause disease. In this review, we revisit the first studies of historical virus genetic material in the 1990s, through to the genomic revolution of recent years. We look at how paleogenomics works for viral pathogens, such as the need for careful precautions against modern contamination and robust computational pipelines to identify and analyze authenticated viral sequences. We discuss the insights into virus evolution which have been gained through paleogenomics, concentrating on three DNA viruses in particular: parvovirus B19, herpes simplex virus 1, and smallpox. As we consider recent worldwide transmission of monkeypox and synthetic biology tools that allow the potential reconstruction of extinct viruses, we show that studying historical and ancient virus evolution has never been more topical.

**Significance**

Ancient viral genomes are an important source of evidence of how viruses evolve in time and space. They provide a record of virus lineages that have gone extinct and help scientists understand changes in virulence or transmissibility. In this review article, we discuss how ancient virus genomes are extracted from ancient remains and how these data are analyzed. We give examples of unique insights into virus evolution that only ancient genomes can give, focusing on parvovirus, herpes simplex virus, and smallpox. Finally, we consider what ancient virus data might tell us about emerging infectious diseases.

## Introduction

Paleogenomics is the study of the genetics of past or extinct populations at a genomic scale, utilizing ancient deoxyribonucleic acid (aDNA) from plants, animals, or pathogens. Paleogenomics and ancient virology are practiced on a range of time spans, ranging from historical samples from the 1970s all the way back to before the Last Glacial period, and have led researchers to scour desiccated rat middens and Siberian permafrost for traces of ancient viruses.

In this review, we explore the history of research into viruses in historical and ancient material and how methodological developments have advanced our ability to identify and authenticate ancient virus sequence data. We then present case studies from three viruses where paleogenomics has made a major contribution to our understanding of virus evolution: parvovirus B19 (B19V), herpes simplex virus 1 (HSV-1), and variola virus (VARV). Finally, we look to the future of paleovirology.

### Why Study Historical and Ancient Viruses?

When we think of the evolutionary history of viruses, we often have questions around how long a given virus has been present in a particular host; whether modern disease presentations are the same as when viruses first emerged into a given population; and whether humans have always been the host of a given virus. For recently emerged zoonoses, we may able to answer these questions with contemporary human, environmental, and animal reservoir samples (Kan et al. 2005; Sharif-Yakan and Kanj 2014). Many viruses have older origins, but also benefit from similar methods, particularly RNA and retroviruses. This is because they evolve fast enough to study in real time, such as influenza, human immunodeficiency viruses (HIV), and severe acute respiratory syndrome coronavirus 2 (SARS-CoV-2). Other viruses, however, are sufficiently slow evolving that they are mutating almost as slowly as the human genome itself (e.g., the herpesviruses; Wertheim et al. 2021). For double-stranded DNA (dsDNA) viruses, the substitution rate is sufficiently slow that even 50 years’ worth of samples may not be enough to adequately calibrate a molecular clock (Havens et al. 2022). Ancient virus genomics can help to bridge the gap, bringing many more viruses in to the “measurably evolving” camp (Biek et al. 2015).

Understanding the pathology caused by viral infections in the past is also challenging because some viruses or viral lineages have gone extinct and are known only from skeletal lesions or historical reports. There are multiple famous examples from the fifteenth to seventeenth centuries of mysterious epidemics from different continents: from north-western Europe, the *Sudor Anglicus* (variously known as the English sweat or the sweating disease) is thought to be a viral pathogen with a mortality rate of up to 50% (Heyman et al. 2018). Its identity remains unknown, as does that of a milder but similar disease known as the Picardy sweat which caused outbreaks 150 years later. Scientists and historians have suggested anthrax and an unknown hantavirus(es) as possible causative agents, but an unknown flavivirus is also a possibility (Heyman et al. 2018). If these diseases were indeed caused by a virus, extinct or extant, paleogenomic analysis of the remains of human victims is our best prospect of identifying the virus responsible—and asking whether any related viruses are still circulating which have a similar disease-causing potential.

In North and Central America, the arrival of European colonizers in what is now Mexico led to multiple outbreaks of cocoliztli, a word which means “pest” in the Nahuatl language (Acuna-Soto et al. 2004). There is considerable debate about whether cocoliztli was one or many pathogens, either in separate waves or as part of a polymicrobial package. Measles, influenza, and smallpox have all been suggested as pathogens which would have led to high mortality among native Mexican populations during the sixteenth century. Ancient DNA is now beginning to shed light on the cocktail of infectious diseases which arrived in the New World in the bodies of European colonists and forcibly transported enslaved Africans (Guzmán-Solís et al. 2021), adding to studies incorporating other lines of evidence. Some of these ancient DNA studies have raised the prospect that cocoliztli was polymicrobial, as pathogens associated with epidemic burial contexts include the bacterium *Salmonella enterica* Paratyphi C (Vågene et al. 2018) and the viruses human B19V and hepatitis B (Guzmán-Solís et al. 2021). B19V is a virus we will be meeting again later in this review. We now know considerably more about its evolution thanks to ancient DNA analysis of strains dating back thousands of years.

Paleogenomics is also able to reveal extinct relatives of known pathogens, such as the identification of Viking-era VARVs, which are related to more recent smallpox-associated VARVs, but not their direct ancestors (Mühlemann et al. 2020). Viral lineage extinction and lineage replacement is a topic on which paleogenomics has made significant contributions to our understanding of patterns of viral diversity, not just for acute or epidemic viruses such as smallpox, but also for latent and chronic viruses such as hepatitis B virus (HBV) and the herpesviruses (Kocher et al. 2021; Guellil et al. 2022).

### Ancient Viral DNA: A Long Road From Pioneering Fragments to ‘Omic Scale Analysis

Ancient DNA virus evidence now exists for plant, animal, and human-specific viruses and includes members of all Baltimore virus classification groups (positive-sense single-stranded ribonucleic acid [+ssRNA], negative-sense single-stranded RNA [−ssRNA], single-stranded RNA-reverse transcribing [ssRNA-RT], double stranded RNA [dsRNA], single-stranded deoxyribonucleic acid [ssDNA], double-stranded DNA [dsDNA], and double-stranded DNA-reverse transcribing [dsDNA-RT]). A summary of all published ancient RNA and DNA discoveries has recently been published elsewhere (Nishimura et al. 2022). Initial genetic evidence came from (reverse transcriptase) polymerase chain reaction (PCR) amplification of small nucleic acid fragments, but now increasingly comes from metagenomic sequence data. Major milestones for virology are summarized in table 1, and it is interesting to note that it is RNA and retroviruses that were the pioneers of the field, despite the poor preservation of ancient RNA in the archeological record, relative to DNA. Perhaps because of this poorer preservation (discussed below), much recent research on ancient viruses at a genomic scale, including tens or hundreds of ancient and historical genomes, has focused on viruses with ssDNA or dsDNA genomes. For this reason, the major focus of this review will be human DNA viruses.

**Table 1:** Virological Milestones in Paleogenomics

Milestone	Virus (Sample Date)	Year	Notes/References
Ancient RNA virus material recovered	Influenza (1918 Common Era [CE])	1997	Taubenberger et al. 1997
Ancient retrovirus material recovered	HIV (1959 CE)	1998	Zhu et al. 1998
Ancient DNA virus material recovered	Human papillomavirus (1568 CE)	2003	Fornaciari et al. 2003
Complete ancient RNA virus genome	Influenza (1918 CE)	2005	Tumpey et al. 2005
Complete ancient DNA virus genome	Hepatitis B virus (∼1680 CE^a^)	2012	Kahila Bar-Gal et al. 2012

a Based on radiocarbon dating.

The earliest studies of ancient viral nucleic acid were typically stored pathology specimens with contemporary diagnostic or symptom data, or from mummies with preserved soft tissue and visible lesions (Fornaciari et al. 2003; Kahila Bar-Gal et al. 2012). Later, screening for ancient pathogens was on the basis of PCR and then quantitative PCR (qPCR) (Fletcher et al. 2003; Worobey et al. 2008), with PCR- and qPCR-based studies following the same strict and evolving standards of authentication and replication as other kinds of ancient nucleic acid research (Calvignac et al. 2008). The last decade has seen a gold rush of ancient viral genomes, and this follows from a further change in methodology in 2011: the increasing transition from qPCR screening of human samples for specific viruses to much more wide-spread use of metagenomic sequencing of human remains and tissue samples (Spyrou et al. 2019; Duchêne et al. 2020).

The success of the last decade in identifying ancient viruses in human remains has been driven not so much by a desire to detect pathogens within human remains, as by the increased use of metagenomic sequencing to acquire whole host genomes at a range of depths. Deeper host metagenomic data sets have been combined with robust computational tools for identifying the most likely taxonomic origin of reads. The combination of richer data, falling costs, improved aDNA extraction techniques, and new computational biology tools has allowed metagenomic sequencing to act as a screening tool for pathogens (box 1). Samples with authentic viral reads can be prioritized for resampling, sequencing at greater depth, or target enrichment—or a combination of these approaches. These population screening approaches have allowed the identification of viruses such as hepatitis B virus (Kocher et al. 2021), VARV (Mühlemann et al. 2020), and adenovirus (Nielsen et al. 2021), which would not necessarily have been specifically screened for in individual remains and which do not leave diagnostic skeletal lesions (box 1). There is even a recent report of Merkel cell polyomavirus aDNA being recovered from the human skin microbiome associated with a 1,500-year-old head louse nit (Pedersen et al. 2022).

Box 1: **Sequencing Ancient Viruses: A Brief Paleogenomic How-To Guide**

Clean excavation, avoiding contamination, and authentication of reads based on damage patterns.

Because authentic ancient virus’ genetic data only represent a tiny fraction of the sequences in human remains, researchers should minimize possible contamination with modern viruses. This is accomplished by following a set of simple but effective guidelines:

− At the excavation site:

i. Handling of the samples with disposable gloves and other protective gear that should be replaced periodically.
ii. Cleaning the samples with a brush and not water.
iii. Using clean tools for samplings.
iv. Storing the samples in a cold and dry place as soon as possible.

− At the experimental laboratory:

i. Performing experimental procedures in clean dedicated facilities, physically separated from facilities working with modern DNA/RNA.
ii. Using positive air pressure to prevent exterior contamination from entering the lab.
iii. Cleaning surfaces, incoming laboratory and archeological material using ultraviolet (UV) light, bleach, and/or detergent.
iv. Wearing full protective gear including masks, goggles, hair covers, disposable overalls, shoes, and at least two layers of disposable gloves, which should be replaced periodically.

− At the bioinformatic laboratory:

i. Screening databases should contain a catalog of artificial sequences to reduce the number of spurious hits against viral species (Wood et al. 2019).
ii. Screened sequences must be further validated using mapping and/or BLAST.
iii. Mapped sequences should display a low average edit distance and an even distribution of coverage.
iv. Sequences verified by mapping should be tested for the presence of aDNA post-mortem damage at the end of the reads (Dabney et al. 2013; Jónsson et al. 2013).

Metagenomics and/or target enrichment

Genetic data retrieved from ancient samples are metagenomic in nature, which may or may not contain virus. The samples are usually first sequenced in a non-targeted and non-specific way, followed by the metagenomic classification of the generated sequences (Duggan et al. 2016; Margaryan et al. 2018; Guzmán-Solís et al. 2021). Capture-based screening is also possible (Schuenemann et al. 2013; Bos et al. 2015). Once a virus of interest has been identified, targeted enrichment of the virus is performed. Genome capture is cost efficient and retrieves a large amount of data (Duggan et al. 2016; Margaryan et al. 2018; Guzmán-Solís et al. 2021).

The challenges of dating

Ancient viral sequences are of great interest because they allow us to date the emergence of viral lineages. For this to be possible, a correlation between genetic changes and samples’ age must exist (temporal signal). Nonetheless, this process is complex, and several factors can affect the dating:

- The number of genetic changes can be underestimated due to purifying selection and site saturation. There is no temporal signal (Duchêne et al. 2014).
- The selection of an inadequate substitution model to date the virus (Duchêne et al. 2014).
- The ancient virus lineage used to calibrate the phylogeny is too divergent from extant strains. The biological features of the ancient strains, the biological relationships with the host, and their evolutionary rates are different (Aiewsakun and Katzourakis 2016).

## Challenges of Ancient Virus Detection

Current methods for studying ancient virus evolution rely on the sequencing of viral nucleic acids from remains, preserved tissues, and other samples. It is therefore important to reflect that we can only sequence what is preserved, detectable, and classifiable. What do we mean by this?

### Preservation

Nucleic acids in human remains degrade and are damaged over time. The damage results from microorganisms and environmental factors (i.e., climate). Strand fragment bases are subject to damage by environmental factors such as UV light, heat, and DNases/RNases, which generates short fragments of genetic material which have often been deaminated in characteristic ways. Cool or cold, ideally dry, conditions with relatively constant temperatures are better for DNA and RNA preservation; warm, wet conditions and high levels of UV rays are worse for preservation. Soil pH also matters (Campos et al. 2012). DNA preservation therefore reduces with increasing time as nucleic acid degradation occurs. Younger human remains tend to yield better-quality aDNA than older remains, including better-quality ancient pathogen nucleic acid as well as host aDNA. dsDNA is normally better preserved than ssDNA (Lindahl 1993) or RNA. However, recovery and successful mapping of single- and double-stranded viral nucleic acid (which may occur within the same virus, such as hepatitis B virus) are variable. This may be due to factors including the library preparation method or the structure of the region in question (guanine-cytosine [GC]-content and presence of repeats) (Guzmán-Solís et al. 2021). All of these factors influence the success of viral sequence recovery and mapping.

In an archeological context, mineralized tissues such as bone and teeth persist longer in the environment than soft tissues. The composition of the bone or tooth provides some protection for nucleic acid against degradation by soil fungi and bacteria or cyanobacteria in fresh and saltwater environments; there is also protection from damage by, for example, UV radiation (Campos et al. 2012). Soft tissue is another source of ancient viral nucleic acids, though it does not preserve for as long as mineralized bone. Sources include chemically preserved human specimens from anatomical or medical collections or naturally or artificially mummified human remains (Taubenberger et al. 1997; Kahila Bar-Gal et al. 2012; Düx et al. 2020). Where soft tissue is preserved, lesions or specific viral pathologies may be macro- or microscopically evident. This can assist in the identification of specimens or remains likely to contain viral nucleic acid or prioritize samples for screening. Preserved soft tissue samples or plants from historical contexts have proved valuable for the preservation of ancient RNA from plant and animal viruses such as 1912 measles (Düx et al. 2020), 1918 influenza (Taubenberger et al. 1997), and some of the earliest recognized HIV cases (Worobey et al. 2016)).

### Detection

When we consider the variety of viruses a human may be infected by during their lifetime, we face almost the full range of virus replication strategies: acute infection (influenza viruses and rhinoviruses), persistent infection (polyomaviruses and papillomaviruses), latent infection (herpesviruses), and genome integration (e.g., retroviruses such as HIV or human herpesviruses 6A and B)—itself a latency strategy. Designing a single assay to detect all the viruses currently infecting an individually would be extremely challenging with current technology; adding the complete infection history would go beyond techniques currently available. Unsurprisingly, paleogenomics acts as a snapshot of some of the pathogens present with a body site or tissue at the time of death, biased by factors such as virus load, genetic material, and differential preservation of host and pathogen genetic material post-mortem.

As previously mentioned, viruses do not leave unambiguous physical evidence of their presence in human bones. The detection of viral aDNA thus relies on the metagenomic screening of tens or hundreds of samples, to be able to detect and retrieve potential viral sequences. In this regard, archeological context can help to assess the existence of an epidemic episode in the past, typically with the presence of human bodies in mass burials or the historical documentation of epidemics. This approach to find samples suitable for screening has been successfully performed for pathogenic bacteria (Bos et al. 2011) and, in recent studies, also for viruses (Guzmán-Solís et al. 2021). Although samples of this type are readily available, the detection of viral sequences is still challenging due to the viruses’ genome preservation and viral tropism to specific tissues that cannot be recovered. In exceptional circumstances, we have access to well-characterized historical samples, usually belonging to medical collections, for which the presence of a virus is almost certain. For example, that is the case for the 1918 influenza pandemic and historical HIV samples (Zhu et al. 1998; Reid et al. 1999). Nonetheless, those cases are rare, and the detection of ancient viruses mainly still relies on massive metagenomic screening of samples.

This is not a problem unique to paleogenomics. For example, in cases of meningitis in adults, an etiological infectious agent is identified in approximately 50% of cases (McGill et al. 2018). A study of pediatric meningitis across five West African countries identified an etiological infectious agent in only 20% of cases (Kwambana-Adams et al. 2020). Whereas some of these variations reflect differences in testing methodologies and patient age group, they are also influenced by local pathogen milieus and the ability to recover pathogen genetic material from the sample sent for testing. Some pathogens cause “hit and run” pathology, where disease occurs long after exposure; it may no longer be possible to isolate the pathogen from the patient, or the pathogen may only be shed in a body site or bodily fluid that is in some sense “removed” from the site of disease. Acute flaccid paralysis, closely associated with enteroviruses (and enterovirus-D68 in particular), is a good example of this, with the virus being rarely found in cerebrospinal fluid (CSF) of affected individuals even though it may be shed in respiratory samples or stool for weeks (Schubert et al. 2019). Thus, the detection of viruses in both living and deceased people is biased by the tissue that is sampled, the virus load in that tissue, and the temporal relationship between infection, disease, and death. Conversely, an infection may be asymptomatic and cause no long-term sequelae or mortality and yet be detectable in ancient human remains.

### Classification

In order to identify viruses from ancient data, the virus reads must look like something we already know, something that is already in a database or with homology to known viral sequences. Viruses do not have regions such as 16S which help to identify new bacteria. Genomic markers are interesting from a metagenomic point of view because they are composed by a variable and highly conserved region, thus allowing for massive non-organism–specific sequence capture in a process known as metabarcoding. Regarding viruses, this means that most of the extant viral diversity has not been recorded (Paez-Espino et al. 2016). The lack of proper characterization of viral diversity in metagenomic databases has deep implications in the study of ancient virus. Current data sets may include reads from pathogens which are still circulating but have not been identified yet, or they may be extinct with no surviving modern relatives. Fortunately, most ancient virus studies rely on shotgun sequencing methods for initial screening; therefore, generated data can be reanalyzed as metagenomic databases are expanded. It is important to remark on the necessity for regular revision of already existing data using updated reference databases, with the intention of minimizing non-necessary resampling. This would allow for a more efficient use of economic resources, saving time for the researchers and preserving samples with a great scientific or cultural value. In that regard, it is worth mentioning the work carried out by the SPAAM community in its AncientMetagenomeDir database (Fellows Yates et al. 2021).

Recent research on finding new RNA viruses by using the RNA-dependent RNA polymerase (RdRp) suggests these tools and approaches may be useful when applied to aDNA/ancient ribonucleic acid (aRNA) (Neri et al. 2022), but efforts towards ancient virus discovery are further complicated by short fragments and damage/degradation. De novo assembly is an important tool in identifying novel viruses, but de novo assembly is often easier/more successful where read lengths are longer (Paszkiewicz and Studholme 2010). Ancient nucleic acid samples contain short fragments which are less suitable for de novo assembly, further complicating efforts to identify potential viral sequences. In addition to that, ancient nucleic samples are usually complex in origin, being a composite of host, host microbiome, and environmental sequences. This mixture could lead to the construction of erroneous sequences during the de novo assembly process (Steinegger and Salzberg 2020).

To bypass the usage of de novo assembly and the lack of marker genes, metagenomic classification software is usually used. Those programs rely on large databases to classify aDNA sequences generated by shotgun sequencing. Examples of two software packages that use different approaches to classify ancient virus are *MALT* and *KrakenUniq* (Breitwieser et al. 2018; Vågene et al. 2018). *MALT* is a software that uses a DNA or protein database to align sequencing data and classify it to a specific taxon or, if shared among different taxa, to its lowest common ancestor (LCA). Because it is an alignment-based method, it is robust to the possible bias induced by aDNA-intrinsic short sequences or aDNA damage (Hübler et al. 2019), but at the expense of needing a prohibitively large amount of computational resources (Vågene et al. 2018; Hübler et al. 2019). On the other hand, *KrakenUniq* is a classifier that relies on exact *k-mer* (subsequences of length *k*) matches against its database to assign a sequence to a specific taxon. It is computationally fast and efficient (Pockrandt et al. 2022), with the additional benefit of being able to tell the number of unique sequence matches (Breitwieser et al. 2018). This can be used to generate an *E*-value to differentiate true from false positives with a low number of reads (Guellil et al. 2018; Borry 2022). In addition to that, *KrakenUniq* is able to process RNA sequences. However, this method does also have some disadvantages, as short sequences, high GC-content, and high aDNA damage can reduce the accuracy of *KrakenUniq* (Breitwieser et al. 2018).

**Fig. 1.**
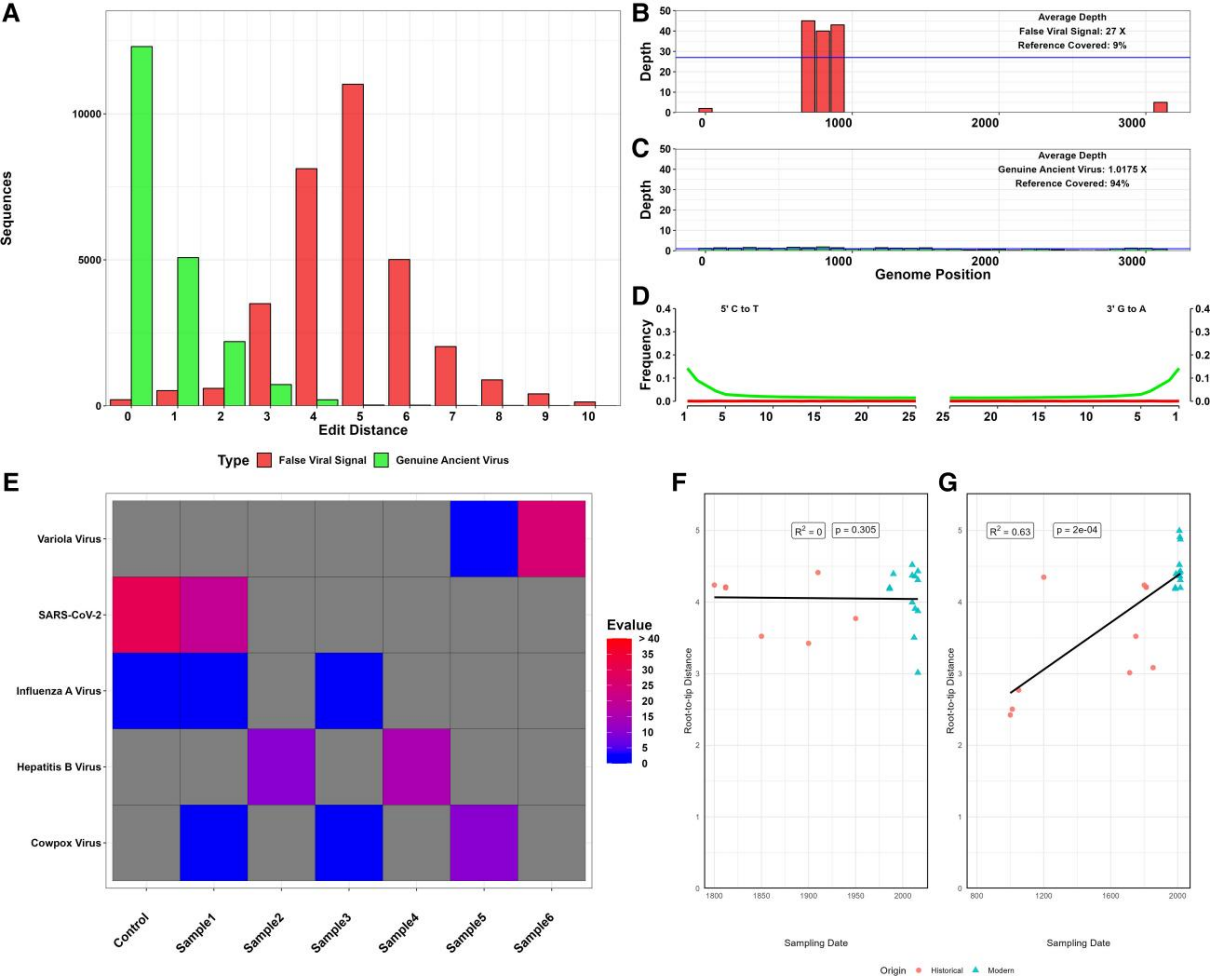
Expected genomic characteristics of genuine ancient viral sequence data, compared with a false viral signal. (*A*) Edit distance distribution of a genuine virus and a false signal. Genuine sequences tend to display lower edit distances (analyzed reads are genetically closer to the assembly) than a false positive signal. (*B* and *C*) Mapped read distribution in a false positive and a genuine viral signal. False positives tend to display an uneven coverage distribution due to the presence of conserved genomic elements or the presence of spuriously assembled sequences in the genome assembly used for mapping, whereas genuine positives are evenly distributed across the reference genome. (*D*) Ancient DNA damage patterns. Genuine sequences present a pattern of deamination at the end of the reads due to post-mortem chemical damage at the DNA molecules; in contrast, false positives do not present those deamination patterns. (*E*) Screening of ancient viruses. Genuine positives are not present in the laboratory controls, and their sequences tend to be unique and evenly distributed along the reference genome. (*F* and *G*) Temporal signal and root-to-tip analysis using ancient virus. Due to factors such as evolutionary pressure, phylogenetic relationships, or site saturation, ancient viral sequence divergence and age are not necessarily correlated.

As we have seen, metagenomic classification software packages are, computationally speaking, expensive and can be affected by the characteristics of the mentioned sequences. It is for that reason that bioinformatic pipelines usually purge aDNA datasets of non-informative sequences which would reduce the performance and accuracy of the classifier (Morfopoulou and Plagnol 2015; Hübler et al. 2019). Examples of those sequences to be purged include sequencing adapters, low-complexity sequences, low-quality sequences, and duplicated sequences. This is also applied to the databases used, which should additionally contain entries for contaminating sequences and the human genome in order to increase the accuracy of the metagenomic classification (Wood et al. 2019). Finally, once the sequences are classified, a final validation step in which the sequences are mapped against the target viral genome is necessary. This is accomplished using a genome aligner software such as *BWA* (Li and Durbin 2009) and, afterwards, further validated by the detection of post-mortem aDNA damage, characterizing read coverage distribution and edit distance distribution, and by the use of what is considered the gold standard technique, *BLAST* (Altschul et al. 1990). Example quality control data are summarized in figure 1. After these necessary hurdles have been overcome, it is possible to progress to the analysis of ancient viral genomes and to begin to draw evolutionary inferences from the data they provide.

## Ancient DNA Viruses, Big and Small

We present here case studies of three human viruses for which several ancient genomes are available and where these data have challenged our understanding of the origins and evolution of circulating viral diversity for each species: B19V, HSV-1, and VARV. Although they are all human-specific DNA viruses, there are significant differences in pathology, epidemiology, and genetics of these viruses. Parvoviruses are some of the smallest human DNA viruses: the genome of B19V is approximately 5.6 kb and the virion is 23–26 nm in size. At the other end of the human DNA scale, VARV's close relative vaccinia virus has virions 360 × 270 nm in size and a genome approximately 190 kb in length; HSV-1 has a genome of 152 kb. HSV-1 infection is lifelong and latent, VARV infection is acute, and B19V infection is normally acute (but there is still debate about whether B19V persists in certain tissues in all healthy infected individuals for life; Norja et al. 2006). Despite these differences, all three have been detected in ancient human remains and have given us fascinating insights into the history of these viruses and us, their hosts.

## Case Study 1: Parvovirus 19

Parvoviruses are small, non-enveloped, ssDNA viruses with genomes of approximately 5 kb. Primate erythroparvovirus 1, better known as B19V, was first identified in 1974. B19V replicates in the bone marrow during primary infection, potentially to very high virus loads, and then “leaks” into other tissue compartments such as the lungs which allows onward transmission to new hosts via respiratory secretions. Disease in healthy children and adults is typically mild and self-limiting; in young children, primary infection is associated with a viral exanthem (Young and Brown 2004). In pregnant women, however, maternal–fetal vertical transmission can occur. This form of fetal infection, especially if it occurs during the first two trimesters of pregnancy, is fatal for the fetus in 2–10% of maternal infections.

Whereas persistent infection is well-documented in some immune-compromised individuals, there is also evidence that in a proportion of healthy individuals, B19V DNA persists in the skin and tonsils, bone marrow, liver, and synovial tissue (Söderlund et al. 1997; Norja et al. 2006). This has enabled the study of the recent evolutionary history of B19V, through a combination of studying contemporary primary infections, persistent virus in adults of a range of ages, and analysis of historical and ancient DNA from human remains and preserved tissues (Norja et al. 2006; Pyöriä et al. 2017; Mühlemann et al. 2018). Samples yielding historical or ancient B19V DNA include teeth, bone marrow, and petrous bone (Mühlemann et al. 2018; Toppinen et al. 2020).

There are currently three recognized B19V genotypes: genotype 1 (distributed worldwide and the predominant European type), genotype 2 (rarely reported in Europe and typically only detected in people born before the mid-1970s; Norja et al. 2006), and genotype 3 (most common in Africa, although more data are needed on its frequency in other regions; Parsyan et al. 2007). Much of the amino acid variability in B19V genomes is concentrated in the VP1 gene, particularly in the unique region which is exposed on the virion surface and is a target for neutralizing antibodies (Modrow and Dorsch 2002). Genotype 1 differs from genotypes 2 and 3 by around 10%, with DNA dissimilarity exceeding 20% in the p6 promoter region (Ekman et al. 2007); genotypes 2 and 3 differ from one another by around 5% (Mühlemann et al. 2018).

Efforts to understand the evolutionary history of B19V have been complicated because of uncertainty over the correct substitution rate to apply to this virus—without a consensus on the rate of the B19V molecular clock, it has been difficult to elucidate the earliest history of this pathogen. Using contemporary sequences, the substitution rate of B19V was estimated to be one of the highest of the ssDNA viruses, in the range 1–2 × 10^−4^ nucleotide substitutions per site per year (subs/site/year), which is comparable to some RNA viruses (Shackelton and Holmes 2006; Parsyan et al. 2007). Substantial variation in rates was seen when comparing B19V sequences derived from plasma and those recovered from biopsies or autopsies (Norja et al. 2008). These studies suggested that the most recent common ancestor (MRCA) of extant B19V diversity was relatively recent: the MRCA of genotype 3 was estimated to be only around 500 years old (Parsyan et al. 2007), whereas the MRCA of genotype 1 as recent as the 1950s (Norja et al. 2008). These studies were at odds with what would be expected from B19V's worldwide distribution and seroprevalence.

Sequencing of ancient B19V genomes, fortuitously discovered as part of metagenomic screening of human remains, has provided important calibration points for calculations of the substitution rate of erythroparvoviruses; in fact, aDNA has transformed our understanding of B19V evolution, coming to conclusions quite different to those derived from circulating and autopsy-derived genomes alone.

Analysis of ancient human remains from Eurasia and Greenland, dating back to 7 thousand years ago (kya), found five genotype 1 and five genotype 2 B19V genomes. B19V genomes from Colonial-era Mexico City (Guzmán-Solís et al. 2021) and one nineteenth century Botocudo individual from eastern Brazil (Dávalos et al. 2022) belonged to genotype 3. These data demonstrate that the genotype structure of B19V is substantially older than previously estimated. By combining all complete modern genomes and ten ancient genomes dating from 500 to 7,000 years old, Mühlemann et al. (2018) estimated that the B19V substitution rate was an order of magnitude slower than previously estimated, at 1.22 × 10^−5^ (1.04 × 10^−5^–1.40 × 10^−5^) subs/site/year. This suggests that the MRCA of B19V is not in the 1800s, but is likely to be around 8000 Before Common Era (BCE). The MRCAs of genotypes 1, 2, and 3 are around 7, 2, and 2.5 thousand years before present, respectively.

Studying ancient B19V genomes also shed light on the evolutionary history of genotype 2. By using ancient and modern genotypes to perform recombination analysis, aDNA confirmed inferences from modern B19V sequences (Shen et al. 2016): genotype 2 is recombinant, formed by an ancient recombination event between types 1 and 3, with the genotype 1 ancestor of portions of the modern genotype 2 genome circulating in humans between 6.9 and 4 kya (Mühlemann et al. 2018).

It was already known that estimating the substitution rate of B19V from plasma samples, or from DNA derived from autopsies and biopsies, led to differing estimations (Norja et al. 2008). By including ancient genomes from Eurasia, Greenland, Mexico, and Brazil (Mühlemann et al. 2018; Guzmán-Solís et al. 2021; Dávalos et al. 2022), we can push the origins of B19V back to the Neolithic (and perhaps beyond). These studies highlight the power of ancient virus DNA for directly dating a pathogen and its different genotypes to a specific place and time, but also the ability of virus aDNA to refine our understanding of the molecular evolution of viral pathogens over a range of time scales.

## Case Study 2: HSV-1

HSV-1 is a dsDNA virus that affects at least two-thirds of the adult human population. Most infections occur during infancy or childhood and are mild or asymptomatic; however, HSV-1 can lead to severe complications, especially in immune-compromised individuals. Following primary infection, the virus becomes latent in sensory neurons. When triggered by psychological or physiological stress, the virus can reactivate resulting in recurrent labial lesions. The host immune system in a healthy individual usually prevents a substantial viremia (Criscuolo et al. 2019).

Modern isolates of HSV-1 go back to 1925 (strain HF) (Flexner and Amoss 1925). Many strains have long and complex histories in tissue culture, including multiple rounds of passage in non-human tissue, leading to artificially inflated mutation rates. This, combined with the relatively high amount of recombination events within strains, makes it difficult to get a temporal signal. For example, from related virus HSV-2, there have been a wide range of estimates of the mutation rate from other studies of HSV-1 (Sakaoka et al. 1994; Firth et al. 2010; Forni et al. 2020).

Because many species have their own species-specific alpha herpes viruses, a co-divergence scenario with anatomically modern humans seems likely; however, recent estimates of modern HSV-1 diversity dated to only the last 7,000 years (Forni et al. 2020). This could be due to a new introduction of the virus or a lineage replacement, as was suggested from data from circulating varicella-zoster virus (VZV, another human alpha herpesvirus) (Weinert et al. 2015).

Currently available ancient HSV-1 genomes come from four individuals from archeological sites across northern Europe, covering the last 2,000 years: a young adult male from an urban medieval hospital cemetery (1350–1450 CE) and an adult female from an early Anglo-Saxon cemetery (500–575 CE) in Cambridgeshire, United Kingdom; an adult male from a burial related to the Nevolino culture (253–530 CE) in Russia; and an adult male from the Netherlands (1600–1700 CE) (Guellil et al. 2022). The individuals harboring these genomes did not show any signs of genetic or physiological susceptibility, but in general had evidence (metagenomic and physical) of periodontal disease. None of the genomes had obvious mutations that would indicate lethal virulence. All the evidence combined suggests these were typical HSV-1 infections.

The estimated age of sampled modern Eurasian HSV-1 diversity is 4.68 (3.87–5.65) kya, and extrapolation of estimated rates to a global data set (including African lineages) points to the age of extant sampled HSV-1 at 5.29 (4.60–6.12) kya, suggesting HSV-1 lineage replacement coinciding with the late Neolithic period and following known Bronze Age migrations. Unfortunately, the age of the recovered genomes precludes answering questions about what was circulating before the lineage replacement (if anything).

It should also be noted that HSV-1 DNA was detected in the deciduous teeth of two children who lived over 30,000 years ago; however, there was insufficient HSV-1 DNA recovered to reconstruct a Pleistocene HSV-1 genome from the teeth. Thus, whereas this is further evidence that HSV-1 is an ancient human pathogen, it is not possible to use this ancient DNA to further reconstruct the deep history of HSV-1, for example, asking whether these ancient HSV-1 infections come from lineages that are extinct today or basal to current Eurasian diversity (Nielsen et al. 2021).

## Case Study 3: VARV

Poxviruses have a reputation as notorious human pathogens, a reputation founded by the morbidity and mortality burden of two members of the orthopox genus: VARV, the causative agent of smallpox, and monkeypox virus (MPXV). Smallpox has been estimated to be the single most deadly infectious disease in human history, because of the cumulative death toll associated with infection and its complications (Krylova and Earn 2020). Indeed, the mortality rate for variola major has been estimated to be between 25% and 40%, and the mortality rate of variola minor to be up to 1% (Henderson 2011; Krylova and Earn 2020). VARV is the only human pathogen to be eradicated by vaccination (declared complete in 1977) and may have much to tell us about emerging disease caused by MPXV.

As a family of viruses, the poxviruses are notable for their genome and viral particle size and their replication within the cytoplasm of infected cells, which is more typical of RNA viruses. Only VARV and molluscum contagiosum virus (MOCV) are obligate human pathogens (Zorec et al. 2018), whereas MPXV can be transmitted by multiple mammals and its natural host species is most likely a rodent (Hutson et al. 2015; Tiee et al. 2018). Much like herpesviruses, poxviruses have multiple mechanisms by which to manipulate infected cells and subvert host immunity. However, unlike human herpesviruses (HHVs), poxviruses are not typically persistent and if natural infection is successfully resolved, protection is lifelong and sterilizing (Hammarlund et al. 2010). During the acute phase of the disease, most individuals with smallpox developed characteristic fluid-filled blisters on their skin; in those who survived initial infection, these blisters would eventually drop off, often leaving behind extensive scarring and sometimes blindness. Dried scabs or blistered skin samples have been preserved in historical and medical collections, providing both visible evidence of smallpox and a potential source of VARV DNA (Duggan et al. 2016; Ferrari et al. 2020). As an extinct virus, laboratory isolates, historical samples such as blisters/infected dried skin, and ancient DNA recovered from bone are the only ways to study the genetic evolution of VARV; ancient samples represent an increasingly important untapped resource.

Because of the relatively distinctive skin rash and disease caused by VARV infection, we are fortunate to have a few different sources of evidence to help us understand the origins of this high-consequence human pathogen. Studies of ancient Egyptian mummies and historical medical textbooks and accounts have suggested that smallpox may have been a human disease since around 2000 BCE, although unambiguous written descriptions of smallpox symptoms only become common around 1600 CE (Thèves et al. 2016). Ancient virus DNA can help us to understand the evolutionary history of VARV by providing another independent line of evidence. It is important to note, however, that different narratives of VARV evolution emerge from studying historical (Duggan et al. 2016; Ferrari et al. 2020) and ancient VARV genomes (Mühlemann et al. 2020), which are not yet fully reconciled.

Studies of VARV genomes derived from historical museum specimens point towards a relatively young age for smallpox. For example, the estimated substitution rates of VARV were estimated by Duggan to be 8.5 × 10^−6^ subs/site/year and by Ferrari to be 10.67 × 10^−6^ subs/site/year. This suggested that VARV diversity sampled between 1944 and 1977 had a relatively recent common ancestor, in the sixteenth to seventeenth century, perhaps as the result of a population bottleneck. These studies of samples from the pre-vaccination era support a view that the diversity of VARV in Europe and North America before the twentieth century was considerably greater than in the post-vaccination period. This would seem difficult to reconcile with evidence of smallpox-like lesions on mummified remains and the presence of ancient written descriptions.

Metagenomic screening of ancient human remains has made an important contribution to how we view VARV genetic evolution. A study spanning the Viking era in northern Europe (600–1050 CE) revealed 11 individuals from this period with VARV DNA sequences preserved in their remains (Mühlemann et al. 2020). Four sets of remains yield near-complete VARV genomes. All the Viking-age VARV sequences fell within a clade of VARV which is distinct from modern and historic strains of VARV which caused smallpox. The presence of this divergent, ancient VARV clade, combined with a substitution rate estimate of 5.16 × 10^−6^ subs/site/year, suggests that different VARV lineages shared a common ancestor around 1,700 years ago.

The Viking-era VARV genomes also had a unique pattern of gene inactivation compared with later VARV strains, some of which imply that ancient VARV had the ability to infect a broader range of hosts than later, historical VARV strains. Other convergent gene inactivations suggest that both modern and ancient VARV lineages had a similar ability to induce a high fever in infected hosts. The phenotype of many of the differentially inactivated VARV genes between lineages is, however, unknown (Mühlemann et al. 2020). This therefore raises the question of whether this Viking-era VARV strains caused the same disease phenotype as historical variola (Alcamí 2020).

Understanding the evolutionary history of VARV, despite its eradication, remains an important scientific goal. Between 2001 and 2018, cases of MPXV increased from 500 to 3,000 cases each year in the Democratic Republic of Congo (DRC), with case fatality rates estimated in a range from below 2% to as high as 11%, depending on MPXV genotype and the HIV status of the infected host (Likos et al. 2005; Beer and Bhargavi Rao 2019). MPXV rates increased 20-fold in the DRC between the 1980s and 2006–2007 and are likely to have increased further since then as the proportion of the population vaccinated against smallpox falls (Rimoin et al. 2010). In 2022, an unprecedented worldwide MPXV outbreak has taken place, with 77,264 laboratory-confirmed cases of monkeypox and 36 associated deaths reported to the WHO between January 1 and October 31, 2022. The majority of cases of MPXV reported outside endemic regions are associated with close bodily contact, primarily sexual contact (Thornhill et al. 2022; World Health Organization 2022). Vaccines developed for VARV are being used to protect at-risk individuals, with the hope of also slowing and preventing MPXV transmission if possible (Gruber 2022).

Studying the ancient evolution of VARV alongside contemporary evolution of MPXV gives virologists vital insights into what makes an *Orthopoxvirus* a successful human pathogen and how changes in particular regions of the genome may be associated with virulence, transmissibility, and host range. It also allows us to ask questions about what impact past selective pressures on the human genome from smallpox-associated mortality have had and whether this allows us to identify individuals at greater risk of severe outcomes following *Orthopoxvirus* infection. Studying recent MPXV evolution may in turn help to generate new hypotheses about early events in VARV evolution, which may become testable if even older VARV aDNA becomes available.

## Going Beyond Ancient Viral Sequences

Ancient samples contain degraded and fragmented portions of viruses which are not replication competent (Ng et al. 2014; Nielsen et al. 2021). A sample that yields ancient virus nucleic acids will not necessarily contain detectable virus protein (Krause-Kyora et al. 2018). Modern molecular virology and immunology tools provide a way to bridge the gap between ancient genotype and in vivo phenotype. Two approaches of note are techniques to create replication competent or incompetent viral clones and pseudotyping studies.

It is possible to create viral clones from synthetic genetic material which are unable to replicate their genomes (replication incompetent), clones which are able to replicate but not proliferate, or clones which replicate and proliferate in a manner akin to wild-type virus (Dudek and Knipe 2006). These techniques are popular in viral vector and vaccine design, but can also be applied to ancient or extinct viruses—controversially including poxviruses (Noyce et al. 2018).

Noyce et al. (2018) synthesized the horsepox genome in a series of fragments from 8 to 32 kb in length; from these fragments, they were able to produce infectious synthetic–chimeric horsepox virus using cells co-infected with a helper virus (Shope fibroma virus). This study has potential bioterrorism implications for extinct viruses with publicly available genome sequences, acting as a proof-of-principle that functional virus particles can be created using synthetic DNA. In the words of Noyce and Evans (2018), “no viral pathogen is likely beyond the reach of synthetic biology.” Others have argued that the tools for this kind of study have existed for some time in molecular biology (Thiel 2018). Ancient pathogen genomics may therefore find itself in unexpected territory in the future, as our ability to recover viral and bacterial genomes of extinct pathogens advances.

There are alternative techniques from molecular biology and viral immunology which allow high-risk pathogens such as Ebola virus to be studied more safely (Watson et al. 2002) and they are likely to be relevant here. For example, pseudotyping studies have been successfully used for SARS-CoV-2, allowing scientists to move rapidly from genome sequence to studies of receptor usage, tissue tropism studies, and antibody neutralization (Hoffmann et al. 2020; Mlcochova et al. 2021) that do not require an isolate of the virus in question. Genetic modification of related or pseudotyped viruses can be used for animal or tissue culture studies (Watson et al. 2002; Liu et al. 2018). Based on methods developed to study extant viruses, immune responses to ancient viral proteins, both cellular and humoral, can be assayed using pseudotyping assays (Mlcochova et al. 2021) or synthesis of viral peptides which mimic natural antigenic stimulation of B and T cells (Houldcroft et al. 2020). These methods give ancient virology scope to study the interactions between a modern human immune system and animal model, which will enrich our understanding of the impact these pathogens had on our ancestors. They will also give us vital information on what impact a similar, re-emergent or novel pathogen might have. Research of this type will need to be undertaken with a view to the most appropriate methods for biosecurity of the virus in question.

## Conclusions

Ancient viral nucleic acid is everywhere: human and animal remains, herbaria specimens, and environmental material such as middens, ice cores, and sediments. Our ability to sequence, classify, and analyze this ancient virome is growing with time, giving us fascinating insights into the tempo and dynamics of viral evolution across a range of time scales. The tools of molecular and synthetic biology are increasingly making it possible to study ancient and extinct viruses in the laboratory alongside circulating viruses. Even as we contemplate the possibilities and pitfalls of virus “de-extinction,” it is important to remember that researching ancient viral pathogens can also give us key insights into emerging infectious diseases. By learning about the viruses which afflicted our ancient ancestors, we may hope to reduce the burden of disease caused by their modern viral relatives.

## Data Availability

Data availability is not applicable to this article as no new data were created or analyzed in this study. For the purpose of open access, the authors have applied a Creative Commons Attribution (CC BY) license to any author accepted manuscript version arising from this submission.

## References

[evad047-B1] Acuna-Soto R , Stahle DW, Therrell MD, Griffin RD, Cleaveland MK. 2004. When half of the population died: the epidemic of hemorrhagic fevers of 1576 in Mexico. FEMS Microbiol Lett. 240:1–5. 15500972 10.1016/j.femsle.2004.09.011 PMC7110390

[evad047-B2] Aiewsakun P , Katzourakis A. 2016. Time-dependent rate phenomenon in viruses ross. J Virol. 90:7184–7195. 27252529 10.1128/JVI.00593-16 PMC4984659

[evad047-B3] Alcamí A . 2020. Was smallpox a widespread mild disease? Science 369:376–377. 32703866 10.1126/science.abd1214

[evad047-B4] Altschul SF , Gish W, Miller W, Myers EW, Lipman DJ. 1990. Basic local alignment search tool. J Mol Biol. 215:403–410. 2231712 10.1016/S0022-2836(05)80360-2

[evad047-B5] Beer EM , Bhargavi Rao V. 2019. A systematic review of the epidemiology of human monkeypox outbreaks and implications for outbreak strategy. PLoS Negl Trop Dis. 13:e0007791. 31618206 10.1371/journal.pntd.0007791 PMC6816577

[evad047-B6] Biek R , Pybus OG, Lloyd-Smith JO, Didelot X. 2015. Measurably evolving pathogens in the genomic era. Trends Ecol Evol. 30:306–313. 25887947 10.1016/j.tree.2015.03.009 PMC4457702

[evad047-B7] Borry M . 2022. A new E-score for KrakenUniq. https://maximeborry.com/ (Accessed March 22, 2023).

[evad047-B8] Bos KI , et al 2011. A draft genome of Yersinia pestis from victims of the black death. Nature 478:506–510. 21993626 10.1038/nature10549 PMC3690193

[evad047-B9] Bos KI , et al 2015. Parallel detection of ancient pathogens via array-based DNA capture. Philos Trans R Soc Lond B Biol Sci. 370:20130375. 25487327 10.1098/rstb.2013.0375 PMC4275883

[evad047-B10] Breitwieser FP , Baker DN, Salzberg SL. 2018. KrakenUniq: confident and fast metagenomics classification using unique k-mer counts. Genome Biol. 19:198. 30445993 10.1186/s13059-018-1568-0 PMC6238331

[evad047-B11] Calvignac S , et al 2008. Ancient DNA identification of early 20th century simian T-cell leukemia virus type 1. Mol Biol Evol. 25:1093–1098. 18296697 10.1093/molbev/msn054

[evad047-B12] Campos PF , et al 2012. DNA in ancient bone—where is it located and how should we extract it? Ann Anat. 194:7–16. 21855309 10.1016/j.aanat.2011.07.003

[evad047-B13] Criscuolo E , et al 2019. Cell-to-cell spread blocking activity is extremely limited in the sera of herpes simplex virus 1 (HSV-1)- and HSV-2-infected subjects. J Virol. 93(11):e00070-19. 10.1128/JVI.00070-19 PMC6532082 30867302

[evad047-B14] Dabney J , Meyer M, Paabo S. 2013. Ancient DNA damage. Cold Spring Harb Perspect Biol. 5:a012567. 23729639 10.1101/cshperspect.a012567 PMC3685887

[evad047-B15] Dávalos DIC , et al 2022. Indigenous peoples in eastern Brazil: insights from 19th century genomes and metagenomes. bioRxiv.

[evad047-B16] Duchêne S , Ho SYW, Carmichael AG, Holmes EC, Poinar H. 2020. The recovery, interpretation and use of ancient pathogen genomes. Curr Biol. 30:R1215–R1231. 33022266 10.1016/j.cub.2020.08.081 PMC7534838

[evad047-B17] Duchêne S , Holmes EC, Ho SYWW. 2014. Analyses of evolutionary dynamics in viruses are hindered by a time-dependent bias in rate estimates. Proc R Soc B Biol Sci. 281:20140732. 10.1098/rspb.2014.0732 PMC4046420 24850916

[evad047-B18] Dudek T , Knipe DM. 2006. Replication-defective viruses as vaccines and vaccine vectors. Virology 344:230–239. 16364753 10.1016/j.virol.2005.09.020

[evad047-B19] Duggan AT , et al 2016. 17th century variola virus reveals the recent history of smallpox. Curr Biol. 26:3407–3412. 27939314 10.1016/j.cub.2016.10.061 PMC5196022

[evad047-B20] Düx A , et al 2020. Measles virus and rinderpest virus divergence dated to the sixth century BCE. Science 368:1367–1370. 32554594 10.1126/science.aba9411 PMC7713999

[evad047-B21] Ekman A , et al 2007. Biological and immunological relations among human parvovirus B19 genotypes 1 to 3. J Virol. 81:6927–6935. 17409158 10.1128/JVI.02713-06 PMC1933287

[evad047-B22] Fellows Yates JA , et al 2021. Community-curated and standardised metadata of published ancient metagenomic samples with AncientMetagenomeDir. Sci Data. 8:1–8. 33500403 10.1038/s41597-021-00816-y PMC7838265

[evad047-B23] Ferrari G , et al 2020. Variola virus genome sequenced from an eighteenth-century museum specimen supports the recent origin of smallpox. Philos Trans R Soc B Biol Sci. 375:20190572. 10.1098/rstb.2019.0572 PMC7702794 33012235

[evad047-B24] Firth C , et al 2010. Using time-structured data to estimate evolutionary rates of double-stranded DNA viruses. Mol Biol Evol. 27:2038–2051. 20363828 10.1093/molbev/msq088 PMC3107591

[evad047-B25] Fletcher HA , Donoghue HD, Holton J, Pap I, Spigelman M. 2003. Widespread occurrence of *Mycobacterium tuberculosis* DNA from 18th-19th century Hungarians. Am J Phys Anthropol. 120:144–152. 12541332 10.1002/ajpa.10114

[evad047-B26] Flexner S , Amoss HL. 1925. Contributions to the pathology of experimental virus encephalitis II. Herpetic strains of encephalitogenic virus. J Exp Med. 41:233–244. 19868984 10.1084/jem.41.2.233 PMC2130935

[evad047-B27] Fornaciari G , et al 2003. Human papillomavirus in a 16th century mummy. Lancet 362:1160. 14550719 10.1016/S0140-6736(03)14487-X

[evad047-B28] Forni D , et al 2020. Recent out-of-Africa migration of human herpes simplex viruses. Mol Biol Evol. 37:1259–1271. 31917410 10.1093/molbev/msaa001

[evad047-B29] Gruber MF . 2022. Current status of monkeypox vaccines. NPJ Vaccines 7:1–3. 35977979 10.1038/s41541-022-00527-4 PMC9385639

[evad047-B30] Guellil M , et al 2018. Genomic blueprint of a relapsing fever pathogen in 15th century Scandinavia. Proc Natl Acad Sci U S A. 115:10422–10427. 30249639 10.1073/pnas.1807266115 PMC6187149

[evad047-B31] Guellil M , et al 2022. Ancient herpes simplex 1 genomes reveal recent viral structure in Eurasia. Sci Adv. 8:2022.01.19.476912. 10.1126/sciadv.abo4435 PMC9328674 35895820

[evad047-B32] Guzmán-Solís AA , et al 2021. Ancient viral genomes reveal introduction of human pathogenic viruses into Mexico during the transatlantic slave trade. Elife 10:e68612. 10.7554/eLife.68612 PMC8423449 34350829

[evad047-B33] Hammarlund E , et al 2010. Antiviral immunity following smallpox virus infection: a case-control study. J Virol. 84:12754–12760. 20926574 10.1128/JVI.01763-10 PMC3004327

[evad047-B34] Havens JL , et al 2022. Phylogeographic analysis reveals an ancient East African origin of human herpes simplex virus 2 dispersal out-of-Africa. Nat Commun. 13:5477. 36115862 10.1038/s41467-022-33214-y PMC9482657

[evad047-B35] Henderson DA . 2011. The eradication of smallpox—an overview of the past, present, and future. Vaccine 29:D7–D9. 22188929 10.1016/j.vaccine.2011.06.080

[evad047-B36] Heyman P , Cochez C, Hukić M. 2018. The English sweating sickness: out of sight, out of mind? Acta Med Acad. 47:102–116. 29957978 10.5644/ama2006-124.221

[evad047-B37] Hoffmann M , et al 2020. SARS-CoV-2 cell entry depends on ACE2 and TMPRSS2 and is blocked by a clinically proven protease inhibitor. Cell 181:271–280.e8. 32142651 10.1016/j.cell.2020.02.052 PMC7102627

[evad047-B38] Houldcroft CJ , et al 2020. Assessing anti-HCMV cell mediated immune responses in transplant recipients and healthy controls using a novel functional assay. Front Cell Infect Microbiol. 10:275. 32670891 10.3389/fcimb.2020.00275 PMC7332694

[evad047-B39] Hübler R , et al 2019. HOPS: automated detection and authentication of pathogen DNA in archaeological remains. Genome Biol. 20:1–13. 31842945 10.1186/s13059-019-1903-0 PMC6913047

[evad047-B40] Hutson CL , et al 2015. Laboratory investigations of African pouched rats (Cricetomys gambianus) as a potential reservoir host species for monkeypox virus Diemert. PLoS Negl Trop Dis. 9:e0004013. 26517724 10.1371/journal.pntd.0004013 PMC4627651

[evad047-B41] Jónsson H , Ginolhac A, Schubert M, Johnson PLF, Orlando L. 2013. Mapdamage2.0: fast approximate Bayesian estimates of ancient DNA damage parameters. Bioinformatics 29:1682–1684. 23613487 10.1093/bioinformatics/btt193 PMC3694634

[evad047-B42] Kahila Bar-Gal G , et al 2012. Tracing hepatitis B virus to the 16th century in a Korean mummy. Hepatology 56:1671–1680. 22610996 10.1002/hep.25852

[evad047-B43] Kan B , et al 2005. Molecular evolution analysis and geographic investigation of severe acute respiratory syndrome coronavirus-like virus in palm civets at an animal market and on farms. J Virol. 79:11892. 16140765 10.1128/JVI.79.18.11892-11900.2005 PMC1212604

[evad047-B44] Kocher A , et al 2021. Ten millennia of hepatitis B virus evolution. Science 374:132. 34618559 10.1126/science.abi5658

[evad047-B45] Krause-Kyora B , et al 2018. Neolithic and medieval virus genomes reveal complex evolution of Hepatitis B. Elife 7:e36666. 29745896 10.7554/eLife.36666 PMC6008052

[evad047-B46] Krylova O , Earn DJD. 2020. Patterns of smallpox mortality in London, England, over three centuries. PLoS Biol. 18:e3000506. 33347440 10.1371/journal.pbio.3000506 PMC7751884

[evad047-B47] Kwambana-Adams BA , et al 2020. Etiology of pediatric meningitis in west Africa using molecular methods in the era of conjugate vaccines against pneumococcus, meningococcus, and haemophilus influenzae type B. Am J Trop Med Hyg. 103:696–703. 32458777 10.4269/ajtmh.19-0566 PMC7410464

[evad047-B48] Li H , Durbin R. 2009. Fast and accurate short read alignment with Burrows-Wheeler transform. Bioinformatics 25:1754–1760. 19451168 10.1093/bioinformatics/btp324 PMC2705234

[evad047-B49] Likos AM , et al 2005. A tale of two clades: monkeypox viruses. J Gen Virol. 86:2661–2672. 16186219 10.1099/vir.0.81215-0

[evad047-B50] Lindahl T . 1993. Instability and decay of the primary structure of DNA. Nature 362:709–715. 8469282 10.1038/362709a0

[evad047-B51] Liu T , et al 2018. A recombinant trivalent vaccine candidate against human adenovirus types 3, 7, and 55. Vaccine 36:2199–2206. 29548605 10.1016/j.vaccine.2018.02.050

[evad047-B52] Margaryan A , et al 2018. Ancient pathogen DNA in human teeth and petrous bones. Ecol Evol. 8:3534–3542. 29607044 10.1002/ece3.3924 PMC5869295

[evad047-B53] McGill F , et al 2018. Incidence, aetiology, and sequelae of viral meningitis in UK adults: a multicentre prospective observational cohort study. Lancet Infect Dis. 18:992–1003. 30153934 10.1016/S1473-3099(18)30245-7 PMC6105576

[evad047-B54] Mlcochova P , et al 2021. SARS-CoV-2 B.1.617.2 delta variant replication and immune evasion. Nature 599:114–119. 34488225 10.1038/s41586-021-03944-y PMC8566220

[evad047-B55] Modrow S , Dorsch S. 2002. Antibody responses in parvovirus B19 infected patients. Pathol Biol. 50:326–331. 12116851 10.1016/s0369-8114(02)00302-4

[evad047-B56] Morfopoulou S , Plagnol V. 2015. Bayesian mixture analysis for metagenomic community profiling. Bioinformatics 31:2930–2938. 26002885 10.1093/bioinformatics/btv317 PMC4565032

[evad047-B57] Mühlemann B , et al 2018. Ancient human parvovirus B19 in Eurasia reveals its long-term association with humans. Proc Natl Acad Sci U S A. 115:7557–7562. 29967156 10.1073/pnas.1804921115 PMC6055166

[evad047-B58] Mühlemann B , et al 2020. Diverse variola virus (smallpox) strains were widespread in northern Europe in the viking age. Science 369(6502), eaaw8977. 10.1126/science.aaw8977 32703849

[evad047-B59] Neri U , et al 2022. Expansion of the global RNA virome reveals diverse clades of bacteriophages. Cell 185:4023–4037.e18. 36174579 10.1016/j.cell.2022.08.023

[evad047-B60] Ng TFF , et al 2014. Preservation of viral genomes in 700-y-old caribou feces from a subarctic ice patch. Proc Natl Acad Sci U S A 111:16842–16847. 25349412 10.1073/pnas.1410429111 PMC4250163

[evad047-B61] Nielsen SH et al 2021. 31,600-year-old human virus genomes support a Pleistocene origin for common childhood infections. bioRxiv

[evad047-B62] Nishimura L , Fujito N, Sugimoto R, Inoue I. 2022. Detection of ancient viruses and long-term viral evolution. Viruses 14:1336. 35746807 10.3390/v14061336 PMC9230872

[evad047-B63] Norja P , et al 2006. Bioportfolio: lifelong persistence of variant and prototypic erythrovirus DNA genomes in human tissue. Proc Natl Acad Sci U S A. 103:7450–7453. 16651522 10.1073/pnas.0602259103 PMC1464359

[evad047-B64] Norja P , Eis-Hübinger AM, Söderlund-Venermo M, Hedman K, Simmonds P. 2008. Rapid sequence change and geographical spread of human parvovirus B19: comparison of B19 virus evolution in acute and persistent infections. J Virol. 82:6427–6433. 18417586 10.1128/JVI.00471-08 PMC2447064

[evad047-B65] Noyce RS , Evans DH. 2018. Synthetic horsepox viruses and the continuing debate about dual use research. PLoS Pathog. 14:e1007025. 30286190 10.1371/journal.ppat.1007025 PMC6171955

[evad047-B66] Noyce RS , Lederman S, Evans DH. 2018. Construction of an infectious horsepox virus vaccine from chemically synthesized DNA fragments. PLoS One 13:e0188453. 29351298 10.1371/journal.pone.0188453 PMC5774680

[evad047-B67] Paez-Espino D , et al 2016. Uncovering Earth's virome. Nature 536:425–430. 27533034 10.1038/nature19094

[evad047-B68] Parsyan A , Szmaragd C, Allain JP, Candotti D. 2007. Identification and genetic diversity of two human parvovirus B19 genotype 3 subtypes. J Gen Virol. 88:428–431. 17251559 10.1099/vir.0.82496-0

[evad047-B69] Paszkiewicz K , Studholme DJ. 2010. De novo assembly of short sequence reads. Brief Bioinform. 11:457–472. 20724458 10.1093/bib/bbq020

[evad047-B70] Pedersen MW , et al 2022. Ancient human genomes and environmental DNA from the cement attaching 2,000-year-old head lice nits. Mol Biol Evol. 39(2), msab351. 10.1093/molbev/msab351 PMC8829908 34963129

[evad047-B71] Pockrandt C , Zimin AV, Salzberg SL. 2022. Metagenomic classification with KrakenUniq on low-memory computers. bioRxiv. 10.21105/joss.04908 PMC10438097 37602140

[evad047-B72] Pyöriä L , et al 2017. Extinct type of human parvovirus B19 persists in tonsillar B cells. Nat Commun. 8(1):14930. 10.1038/ncomms14930 PMC5382274 28374737

[evad047-B73] Reid AH , Fanning TG, Hultin JV, Taubenberger JK. 1999. Origin and evolution of the 1918 ‘Spanish’ influenza virus hemagglutinin gene. Proc Natl Acad Sci U S A. 96:1651–1656. 9990079 10.1073/pnas.96.4.1651 PMC15547

[evad047-B74] Rimoin AW , et al 2010. Major increase in human monkeypox incidence 30 years after smallpox vaccination campaigns cease in the Democratic Republic of Congo. Proc Natl Acad Sci U S A. 107:16262–16267. 20805472 10.1073/pnas.1005769107 PMC2941342

[evad047-B75] Sakaoka H , et al 1994. Quantitative analysis of genomic polymorphism of herpes simplex virus type 1 strains from six countries: studies of molecular evolution and molecular epidemiology of the virus. J Gen Virol. 75(Pt 3):513–527. 8126449 10.1099/0022-1317-75-3-513

[evad047-B76] Schubert RD , et al 2019. Pan-viral serology implicates enteroviruses in acute flaccid myelitis. Nat Med. 25:1748–1752. 31636453 10.1038/s41591-019-0613-1 PMC6858576

[evad047-B77] Schuenemann VJ , et al 2013. Genome-wide comparison of medieval and modern Mycobacterium leprae. Science 341:179–184. 23765279 10.1126/science.1238286

[evad047-B78] Shackelton LA , Holmes EC. 2006. Phylogenetic evidence for the rapid evolution of human B19 erythrovirus. J Virol. 80:3666–3669. 16537636 10.1128/JVI.80.7.3666-3669.2006 PMC1440363

[evad047-B79] Sharif-Yakan A , Kanj SS. 2014. Emergence of MERS-CoV in the Middle East: origins, transmission, treatment, and perspectives. PLoS Pathog. 10:e1004457. 25474536 10.1371/journal.ppat.1004457 PMC4256428

[evad047-B80] Shen H , Zhang W, Wang H, Shao S. 2016. Identification of recombination in the NS1 and VPs genes of parvovirus B19. J Med Virol. 88:1457–1461. 26756922 10.1002/jmv.24471

[evad047-B81] Söderlund M , et al 1997. Persistence of parvovirus B19 DNA in synovial membranes of young patients with and without chronic arthropathy. Lancet 349:1063–1065. 9107245 10.1016/S0140-6736(96)09110-6

[evad047-B82] Spyrou MA , Bos KI, Herbig A, Krause J. 2019. Ancient pathogen genomics as an emerging tool for infectious disease research. Nat Rev Genet. 20(6):323-40. 10.1038/s41576-019-0119-1 PMC7097038 30953039

[evad047-B83] Steinegger M , Salzberg SL. 2020. Terminating contamination: large-scale search identifies more than 2,000,000 contaminated entries in GenBank. Genome Biol. 21:1–12. 10.1186/s13059-020-02023-1 PMC7218494 32398145

[evad047-B84] Taubenberger JK , Reid AH, Krafft AE, Bijwaard KE, Fanning TG. 1997. Initial genetic characterization of the 1918 ‘Spanish’ influenza virus. Science 275:1793–1796. 9065404 10.1126/science.275.5307.1793

[evad047-B85] Thèves C , Crubézy E, Biagini P. 2016. History of smallpox and its spread in human populations. Microbiol Spectr. 4(4):PoH-0004-2014. 10.1128/microbiolspec.PoH-0004-2014 27726788

[evad047-B86] Thiel V . 2018. Synthetic viruses—anything new? PLoS Pathog. 14:e1007019. 30286176 10.1371/journal.ppat.1007019 PMC6171941

[evad047-B87] Thornhill JP , et al 2022. Monkeypox virus infection in humans across 16 countries—April–June 2022. N Engl J Med. 387:679–691. 35866746 10.1056/NEJMoa2207323

[evad047-B88] Tiee MS , Harrigan RJ, Thomassen HA, Smith TB. 2018. Ghosts of infections past: using archival samples to understand a century of monkeypox virus prevalence among host communities across space and time. R Soc Open Sci. 5:171089. 29410823 10.1098/rsos.171089 PMC5792900

[evad047-B89] Toppinen M , et al 2020. The landscape of persistent human DNA viruses in femoral bone. Forensic Sci Int Genet. 48:102353. 32668397 10.1016/j.fsigen.2020.102353

[evad047-B90] Tumpey TM , et al 2005. Characterization of the reconstructed 1918 Spanish influenza pandemic virus. Science 310:77–80. 16210530 10.1126/science.1119392

[evad047-B91] Vågene ÅJ , et al 2018. Salmonella enterica genomes from victims of a major sixteenth-century epidemic in Mexico. Nat Ecol Evol. 2:520–528. 29335577 10.1038/s41559-017-0446-6

[evad047-B92] Watson DJ , Kobinger GP, Passini MA, Wilson JM, Wolfe JH. 2002. Targeted transduction patterns in the mouse brain by lentivirus vectors pseudotyped with VSV, Ebola, Mokola, LCMV, or MuLV envelope proteins. Mol Ther. 5:528–537. 11991743 10.1006/mthe.2002.0584

[evad047-B93] Weinert LA , et al 2015. Rates of vaccine evolution show strong effects of latency: implications for varicella zoster virus epidemiology. Mol Biol Evol. 32:1020–1028. 25568346 10.1093/molbev/msu406 PMC4379407

[evad047-B94] Wertheim JO , et al 2021. Discovery of novel herpes simplexviruses in wild gorillas, bonobos, and chimpanzees supports zoonotic origin of HSV-2. Mol Biol Evol. 38(7):2818-30 10.1093/molbev/msab072 PMC8233514 33720357

[evad047-B95] Wood DE , Lu J, Langmead B. 2019. Improved metagenomic analysis with Kraken 2. Genome Biol. 20:257. 31779668 10.1186/s13059-019-1891-0 PMC6883579

[evad047-B96] World Health Organization . 2022. Multi-country outbreak of monkeypox, External situation report #9. [updated 2022 Nov 2; cited 2022 Nov 8]. WHO Situat. Rep. https://www.who.int/publications/m/item/multi-country-outbreak-of-monkeypox–external-situation-report–9—2-november-2022.

[evad047-B97] Worobey M , et al 2008. Direct evidence of extensive diversity of HIV-1 in Kinshasa by 1960. Nature 455:661–664. 18833279 10.1038/nature07390 PMC3682493

[evad047-B98] Worobey M , et al 2016. 1970s and ‘patient 0’ HIV-1 genomes illuminate early HIV/AIDS history in North America. Nature 539:98–101. 27783600 10.1038/nature19827 PMC5257289

[evad047-B99] Young NS , Brown KE. 2004. Parvovirus B19. N Engl J Med. 350:586–597. 14762186 10.1056/NEJMra030840

[evad047-B100] Zhu T , et al 1998. An African HIV-1 sequence from 1959 and implications for the of the epidemic. Nature 391:594–597. 9468138 10.1038/35400

[evad047-B101] Zorec T , et al 2018. New insights into the evolutionary and genomic landscape of molluscum contagiosum virus (MCV) based on nine MCV1 and six MCV2 complete genome sequences. Viruses 10:586. 30373153 10.3390/v10110586 PMC6266040

